# Glandular Trichomes and Essential Oil of *Thymus quinquecostatus*

**DOI:** 10.1155/2013/387952

**Published:** 2013-10-23

**Authors:** Ping Jia, Hanzhu Liu, Ting Gao, Hua Xin

**Affiliations:** ^1^College of Life Sciences, Qingdao Agricultural University, Qingdao 266109, China; ^2^University Key Laboratory of Plant Biotechnology in Shandong Province, Qingdao 266109, China

## Abstract

The distribution and types of glandular trichomes and essential oil chemistry of *Thymus quinquecostatus* were studied. The glandular trichomes are distributed on the surface of stem, leaf, rachis, calyx and corolla, except petiole, pistil and stamen. Three morphologically distinct types of glandular trichomes are described. Peltate trichomes, consisting of a basal cell, a stalk cell and a 12-celled head, are distributed on the stem, leaf, corolla and outer side of calyx. Capitate trichomes, consisting of a unicellular base, a 1–2-celled stalk and a unicellular head, are distributed more diffusely than peltate ones, existing on stem, leaf, rachis and calyx. Digitiform trichomes are just distributed on the outer side of corolla, consisting of 1 basal cell, 3 stalk cells and 1 head cell. All three types of glandular trichomes can secrete essential oil, and in small capitate trichomes of rachis, all peltate trichomes and digitiform trichomes, essential oil is stored in a large subcuticular space, released by cuticle rupture, whereas, in other capitate trichomes, essential oil crosses the thin cuticle. The essential oil of *T. quinquecostatus* is yellow, and its content is highest in the growth period. 68 constituents were identified in the essential oils. The main constituent is linalool.

## 1. Introduction

There are external and internal secretory structures identified based on their positions in the plant organs. Glandular trichomes, belonging to external secretory structures, can secrete various materials, such as essential oil, which play an important role in plant growth and development. Therefore, studies on the glandular trichomes attracted many scholars these years. Due to their abundant physiological activities, plant essential oils are used in various fields, especially the *Thymus* species essential oil. The *Thymus* species are well-known sources of antimicrobial essential oils and their compositions vary tremendously in different plant species.* Thymus quinquecostatus* is a scrubby subshrub of *Thymus *Lamiaceae, found on mountain slopes and the hilly seaside. Its essential oil contains many constituents [1], used for cosmetic fragrance and flavouring essence. Essential oil of *Thymus quinquecostatus* is often effective as an antibiosis, an insecticide, an antitumor substance, and so on [2, 3]. As a result, it has been widely used in the pharmaceutical industry, the cosmetic industry, the food industry, and so forth [4, 5]. In some regions of China, people make leaves of *Thymus quinquecostatus* into beverage to prevent sunstroke or burn them to keep off mosquitoes. Moreover, *Thymus quinquecostatus* is a high-quality pasture. There is not much information about glandular trichomes on leaves of *Thymus quinquecostatus* [6]. Therefore, the aim of this research is to conduct a systematic study on glandular trichomes and essential oil of *Thymus quinquecostatus.*

## 2. Materials and Methods

### 2.1. Plant Materials

From April to October of 2011, leaves, stems, rachis, and flowers of *T. quinquecostatus* were collected from Laoshan Mountains in Qingdao, China.

### 2.2. Methods

#### 2.2.1. Stereomicroscope

The secretion of leaves, flowers, stems, and rachis was observed with BH-2 OLYMPUS stereomicroscope.

#### 2.2.2. Light Microscopy (LM)

Whole flowers and 5 mm^2^ small pieces of the materials (leaf, stem, and rachis) were fixed in FAA and embedded in paraffin. 8–10 *μ*m pieces were sectioned with a LEICA RM2126RT Rotary Microtome. All materials had transverse sections. The sections were stained with ferroalumen-hematoxylin and observed with an OLYMPUS BH-2 microscope.

#### 2.2.3. Scanning Electron Microscopy (SEM)

Leaf, stem, rachis, calyx, corolla, stamen, and pistil samples were fixed in glutaraldehyde and paraformaldehyde. After being dehydrated and dried at critical point, they were coated with gold in the vacuum evaporator. Finally, the preparations were observed and photographed with a KYKY-2800B SEM.

#### 2.2.4. Extraction and Analysis of Essential Oil

Aboveground parts of* T. quinquecostatus *were separately collected in May (growth period), July (flowering period), and October (nearly withered period) of 2011. Essential oils were obtained by steam distillation of them. The compositions of oil fraction were analyzed by gas chromatography/mass spectrometry.

## 3. Results

### 3.1. Types, Distribution, and Structure of Glandular Trichomes

#### 3.1.1. Leaf

**Figure 1:**
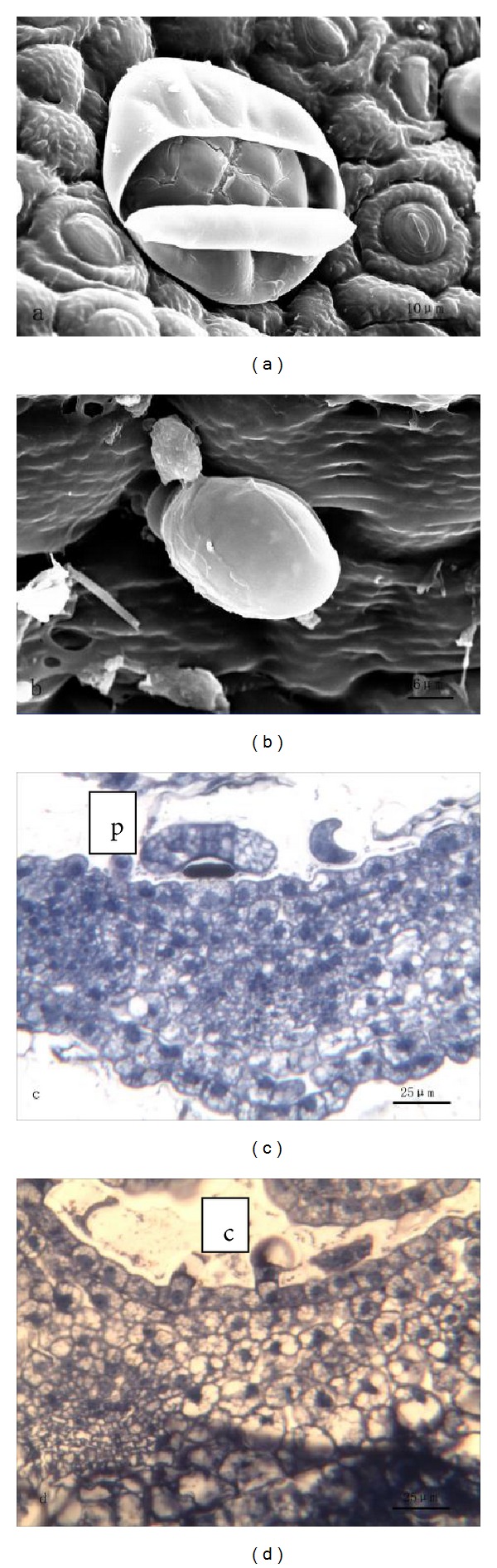
((a), (b)) SEM micrographs showing the morphology of glandular trichomes on the leaf of *T. quinquecostatus.* (a) A peltate trichome. (b) A capitate trichome. ((c), (d)) Transverse sections of *T. quinquecostatus* leaf. (c) A peltate trichome (p). (d) A capitate trichome (c).

A leaf of *T. quinquecostatus* consists of a petiole and a lamina. There is not any glandular trichome on the surface of a petiole. Peltate and capitate glandular trichomes are distributed both on the upper epidermis and lower epidermis of lamina. These two types are very different in morphology and structure. The peltate trichome consists of a basal cell, a stalk cell, and a multicellular head (Figure 1(c)). The head has 12 secretory cells forming two cycles, which includes 4 central and 8 peripheral cells (Figure 1(a)). The capitate trichome consists of a basal cell, a stalk cell, and a unicellular head (Figure 1(d)). Along with growth, peltate hairs sank gradually, while capitate hairs remained unchanged. With the help of a stereomicroscopy, researchers found that abundant essential oil existed in the pit, formed by the sinking of peltate hairs, and on the head of the capitate glandular hair. In the capitate trichomes, secretion was extruded through the cuticle (Figure 1(b)), while in the peltate ones, the secretion kept on accumulating in the subcuticular space and finally released by the cuticle rupture (Figure 1(a)).

#### 3.1.2. Stem

**Figure 2:**
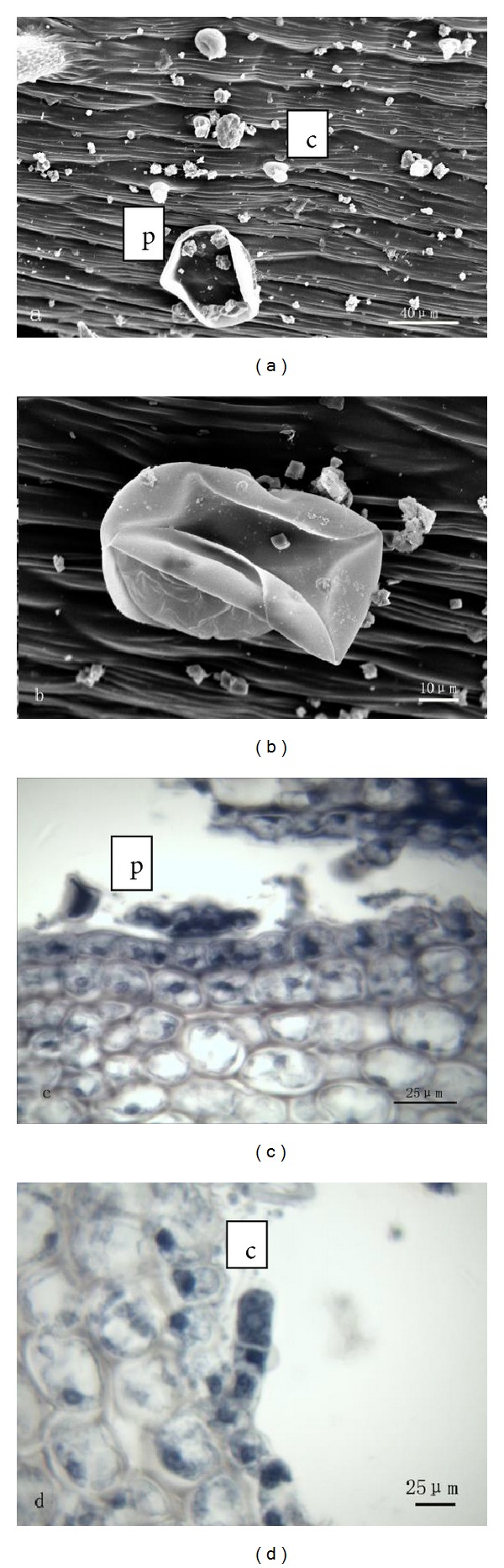
((a), (b)) SEM micrographs showing the morphology of glandular trichomes on stem of *T. quinquecostatus. *(a) Peltate trichomes (p) and capitate ones (c). (b) A peltate trichome. ((c), (d)) Transverse sections of *T. quinquecostatus* stem. (c) A peltate trichome (p). (d) A capitate trichome (c).

Both peltate and capitate glandular trichomes are distributed on the surface of the stem (Figure 2(a)). The peltate trichome consists of a basal cell, a stalk cell, and a multicellular head (Figure 2(c)). The head, which looks like that of the peltate one on leaves, consists of 12 secretory cells, forming two cycles. But, unlike the peltate hairs on leaves, these peltate hairs did not sink but remained in their normal positions. Secretion was released by the cuticle rupture in the peltate trichome (Figure 2(b)). The capitate trichome consists of a basal cell, two stalk cells, and a unicellular head (Figure 2(d)). Secretion was extruded through its cuticle. Essential oil were observed to be secreted from heads of two types of glandular trichomes with stereomicroscope.

#### 3.1.3. Rachis

**Figure 3:**
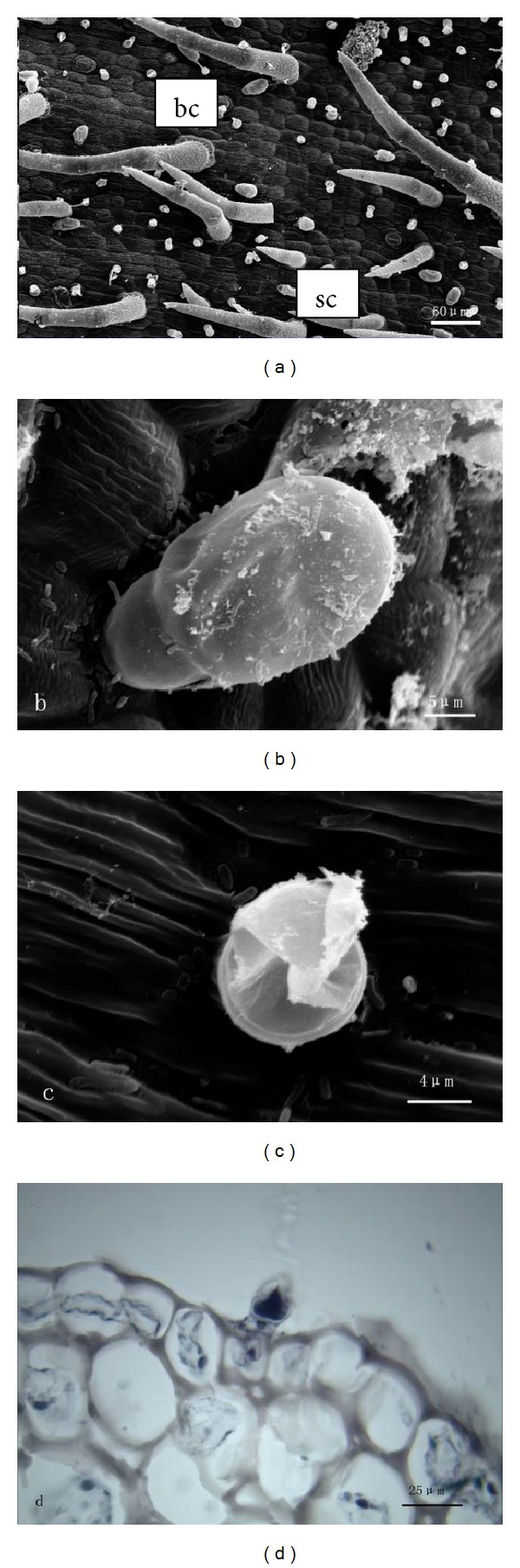
((a)–(c)) SEM micrographs showing the morphology of glandular trichomes on rachis of *T. quinquecostatus.* (a) The surface of rachis, showing big capitate trichomes (bc) and small capitate trichomes (sc). (b) A big capitate trichome. (c) A small capitate trichome. (d) Transverse section of rachis of *T. quinquecostatus*, showing a capitate trichome.

Only capitate glandular trichomes are distributed on the surface of rachis (Figures 3(a) and 3(b)). There are two different morphological types of capitate hairs, the big and the small. But the structure of the two types of hairs is the same; they both consist of a basal cell, a stalk cell, and a head cell (Figure 3(d)). When glandular trichomes matured, essential oil of big capitate trichomes would be extruded through their cuticle. In small capitate trichomes, secretion was released through cuticle rupture of head cells (Figure 3(c)). The essential oil on the surface of head cells was observed with stereomicroscopy.

#### 3.1.4. Calyx

**Figure 4:**
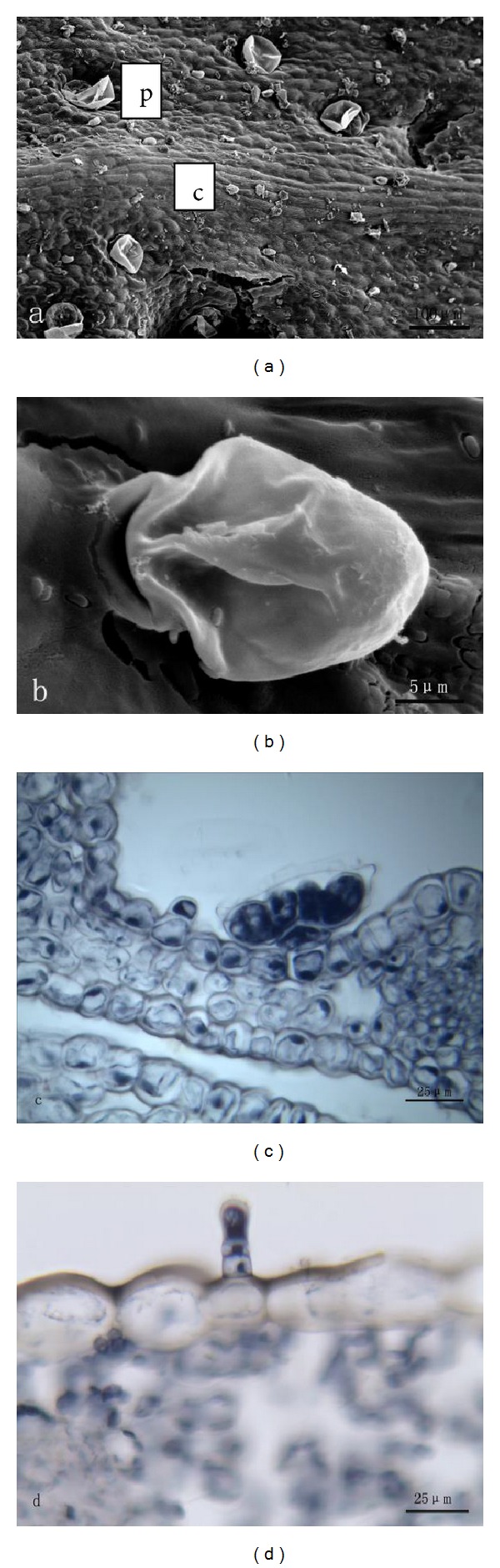
((a), (b)) SEM micrographs showing the morphology of glandular trichomes on outer side of calyx of *T. quinquecostatus.* (a) Peltate trichomes (p) and capitate trichomes (c). (b) A capitate trichome. ((c), (d)) Transverse sections of *T. quinquecostatus *calyx. (c) A peltate trichome. (d) A capitate trichome.

*(1) The Outer Side of Calyx. *Two types of glandular trichomes are distributed on the outside surface of calyx, namely, peltate ones and capitate ones (Figure 4(a)). Like that of leaves, peltate trichome has a basal cell, a short stalk cell, and a large head (Figure 4(c)), while the head presents a peltate shape. The head of the peltate one has 12 secretory cells forming two cycles, which includes 4 central and 8 peripheral cells. Along with the growth, peltate glandular trichomes sank gradually. Essential oil was observed in sunken pit. Capitate trichome consists of a basal cell, two stalk cells, and a unicellular head (Figure 4(d)). Researchers found that the essential oil existed on the head. In the capitate trichomes, secretion was extruded through the cuticle (Figure 4(b)), while in the peltate ones, the secretion was released by cuticle rupture (Figure 4(a)).

**Figure 5:**
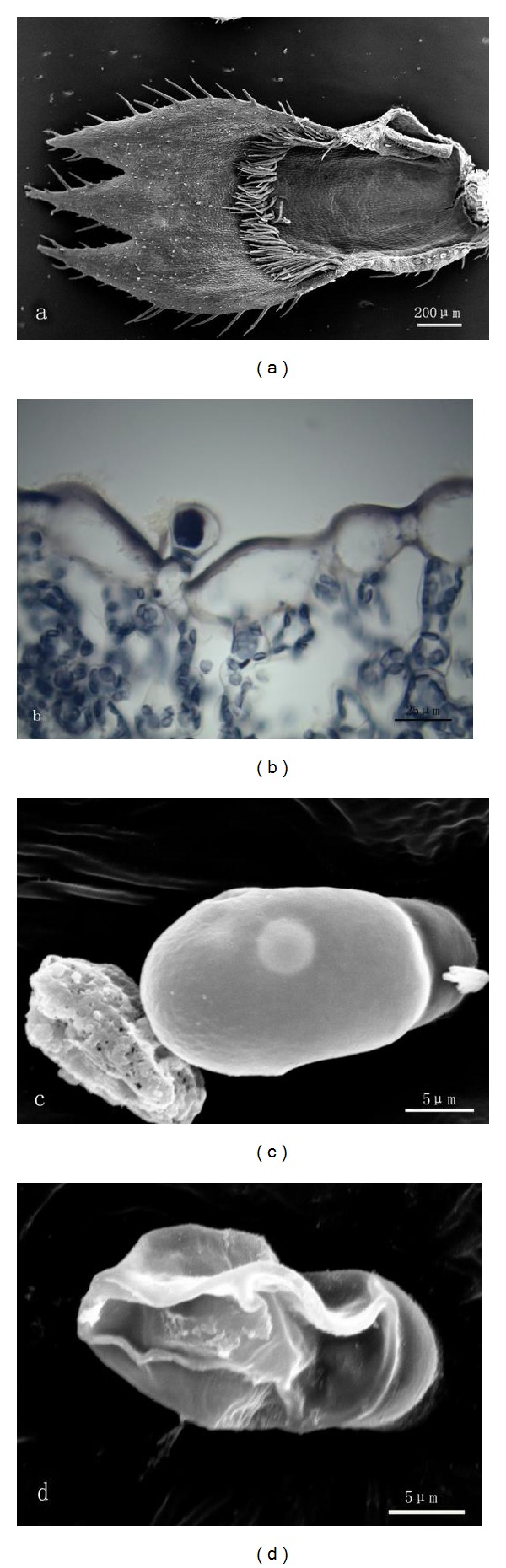
((a), (c), and (d)) SEM micrographs showing the morphology of capitate trichomes on inner side of calyx. (a) The distribution of capitate trichomes. (b) Transverse section of *T. quinquecostatus *calyx, showing a capitate trichome. ((c), (d)) Changes of a capitate trichome.

*(2) The Inside Surface of Calyx*. Unlike the glandular trichomes on the outer side of calyx, only one type of trichome, which is capitate trichome, is distributed on the inner side. These trichomes are only distributed on the upper part of calyx, while on the lower part, no glandular trichome exists (Figure 5(a)). The capitate trichome consists of a basal cell, a stalk cell, and a unicellular head cell. The head cell is oval-shaped (Figure 5(b)). As the secretion was released, the cuticle of capitate trichome becomes shriveled (Figures 5(c) and 5(d)). Essential oil was observed on the head of capitate glandular hair with a stereomicroscopy.

#### 3.1.5. Corolla

**Figure 6:**
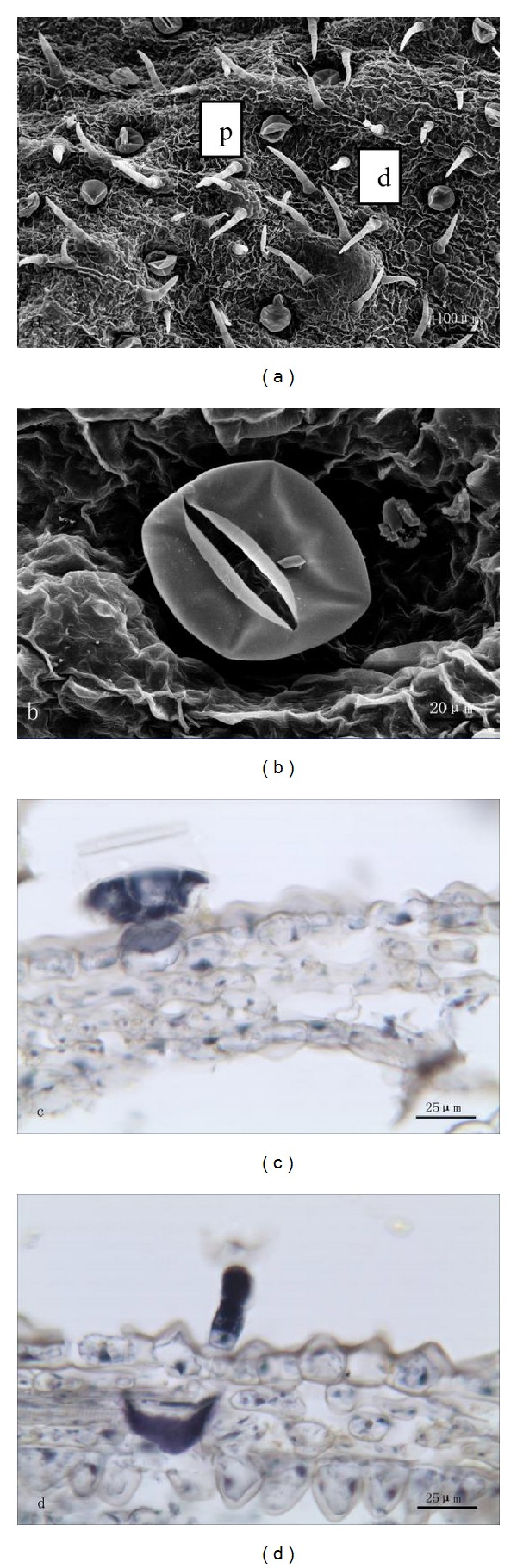
((a), (b)) SEM micrographs showing the morphology of glandular trichomes on outer side of corolla of *T. quinquecostatus.* (a) Peltate trichomes (p) and digitiform ones (d). (b) A peltate trichome. ((c), (d)) Transverse sections of *T. quinquecostatus *corolla. (c) A peltate trichome. (d) A digitiform trichome.

Two types of glandular trichomes, peltate and digitiform ones, exist on the outer side of corollas (Figure 6(a)). No glandular trichomes exist on the inner side of corolla. Like the peltate one on the leaves, the peltate glandular trichome also consists of a basal cell, a stalk cell, and a large disc-like head (Figure 6(c)). And the head consists of 12 secretory cells which include 4 central and 8 peripheral cells. When it is matured, the secretion will be released by the cuticle rupture (Figure 6(b)). The digitiform trichome, which does not show a clear distinction between the apical glandular cell and the subsidiary cells, consists of five cells totally, which includes one basal cell, three stalk cells, and one head cell (Figure 6(d)). The secretions got released by the cuticle rupture. Essential oil existing on both types of glandular hairs was observed with stereomicroscopy.

#### 3.1.6. Stamen and Pistil

*Thymus quinquecostatus* consists of 4 stamens and 1 pistil. No glandular trichome was observed on surface of stamens and stigma, style, and ovary of pistil 1.

### 3.2. Essential Oil

Although essential oil in different periods of *T. quinquecostatus* is yellow, essential oil content is decreased with the growth.  Table 1 shows their contents, with the highest one appearing in the growth period.

Table 2 shows the constituents of essential oil of *T. quinquecostatus* in different periods. There were 68 constituents identified in the essential oils, 46 in growth period, 46 in flowering period, and 39 in nearly withered period. The main constituent was linalool.

## 4. Discussion

The essential oil, which is produced by glandular trichomes, is one of the characteristic features of the Lamiaceae family. Glandular trichomes of this family are among the most investigated secretory structures. The types and distribution of glandular trichomes are diverse among different genus or species plants of Labiatae, so the glandular trichome is regarded as a basis of classification of Lamiaceae. *Salvia argentea* has two types of glandular trichomes, peltate and capitate ones. Capitate glandular trichomes are distributed on all aerial organs, but the number of basal cells, stalk cells and head cells is varied depending on different types of capitate glandular hairs; peltate glandular hairs are distributed on all aerial organs except for petiole; moreover, central and circumambient cell numbers are different depending on different types of peltate glandular hairs [7]. Five types of glandular trichomes are described in *Plectranthus ornate*. Peltate trichomes are distributed on the leaf abaxial surface, stamens, and carpel. Digitiform glandular trichomes and capitate ones are distributed on both sides of leaves. Capitate glandular trichomes consist of two types, namely, long-stalked capitate trichomes and short-stalked ones; conoidal trichomes are distributed on the surface of calyx and corolla; besides, capitate trichomes are also distributed on the calyx and corolla surface [8]. Capitate trichomes also exist on fruit surface of some species of *Lycopus *[9]. By researching on some plants of Lamioideae Lamiaceae, glandular trichomes can be divided into 3 types, namely, peltate glandular trichomes, small capitate ones, and large capitate ones. These trichomes are greatly distinct in different species or on leaf, calyx, and corolla [10]. On the basis of the results, it is found that glandular trichomes are distributed on leaves, stems, rachis, calyx, and corolla of *Thymus quinquecostatus,* except for its petiole, stamens, and pistil. Glandular trichomes are of three types, namely, peltate trichomes, capitate ones, and digitiform ones. Peltate trichomes are distributed on adaxial and abaxial surface of leaves, stems, outer side of calyx, and corolla, and they all consist of 1 basal cell, 1 stalk cell, and 1 12-celled head. Capitate trichomes are distributed on adaxial and abaxial surface of leaves, stems, rachis, and outer side and inner side of calyx, but capitate trichomes on different organs are diverse, consisting of 1 basal cell, 1-2 stalk cells, and 1 head cell. Digitiform trichomes are just distributed on outer side of corolla, forming 1 basal cell, 3 stalk cells, and 1 head cell.

The glandular hairs of Lamiaceae likely release various secretions, such as the essential oil and polysaccharide. When trichomes got matured, essential oil could be found on all of the glandular trichomes on aerial organs of* Thymus quinquecostatus*. Besides essential oil, what else trichomes could secrete needs further study. There are three secretory pathways of releasing essential oil; some are extruded through the cuticle; some formed subcuticular space and finally were released through cuticle rupture; some formed a small pore and were released through the apical pore of secretory cells [10–12]. In our research, there are two secretory pathways of glandular trichomes of* Thymus quinquecostatus*; in small capitate trichomes of rachis, all peltate trichomes, and digitiform ones, essential oil accumulates in the subcuticular space between them and is released through the cuticle rupture. Cuticle of other capitate trichomes on different organs is complete, and essential oil is released through the cuticle.

The essential oil is highly complex chemical compounds. The content and composition of it vary depending on season, growing conditions, and plant species. Carvacrol and thymol are the main constituents in some species of *Thymus*, such as *T. quinquecostatus *(in Korean), *T. magnus*,* T. transcaspicus,* and *T. persicu *[13–15]. In our research, the main constituent of* T. quinquecostatus* essential oil is linalool, and its content is about 40% in growth period, flowering period, or nearly withered period. But, essential oil in flowering period has a particular compound, borneol, and its content is about 15%. Carvacrol is only about 0.1% in three periods, and thymol has not been detected. The main constituents of essential oil may be different according to the growing conditions of plants. In general, the highest content of essential oil appears at the growth period. In this period, many leaves grew. So a lot of glandular trichomes on leaf surface were produced. High essential oil content is related to much secretion of glandular trichomes.

## Figures and Tables

**Table 1:** Content of essential oil of *T. quinquecostatus* in different periods.

Period	Content (%)
Growth period	1.40
Flowering period	1.22
Nearly withered period	1.00

**Table 2:** Constituents of essential oil of *T. quinquecostatus* in different periods.

Compound	Content (%)		
	Growth period	Flowering period	Nearly withered period
Tricyclene	0.03	0.03	0.04
Thujene	0.03	0.03	0.05
*α*-Pinene	0.53	/	/
Camphene	1.12	0.93	1.56
1-Octen-3-ol	0.51	0.84	1.32
2-Pentylfuran	/	0.05	/
*β*-Myrcene	/	0.46	/
*β*-Pinene	0.20	0.23	0.38
2-Propylpyridine	0.28	/	/
(E,E)-2,4-Nonadienal	/	/	0.05
Terpinolene	/	/	0.05
(+)-2-Carene	0.07	/	/
*β*-Cymene	0.20	0.39	/
Eucalyptol	0.50	2.24	1.50
D-Limonene	0.89	1.02	1.41
*trans*-*β*-Ocimene	0.07	0.06	0.11
*γ*-Terpinene	0.21	0.16	0.18
1-Nonen-3-ol	/	0.06	/
Linalool	40.31	39.1	45.44
D-Camphor	0.14	/	0.16
Carvacrol	0.08	0.13	0.12
4-Methoxystyrene	0.06	0.23	0.13
*trans*-2-Nonenal	/	0.05	/
Borneol	/	14.58	/
l-4-Terpineneol	1.86	2.14	2.56
*α*-Terpineol	0.53	0.96	0.70
cis-Piperitol	0.04	0.07	0.05
Nerol	0.11	/	0.08
D-Carvone	0.04	0.08	0.07
Cyclodecane	/	/	0.13
Geraniol	0.12	/	/
2-Cyclohexen-1-ol	/	0.13	/
Nonanoic acid	/	0.15	/
(E,E)-2,4-Decadienal	/	0.05	/
Linalyl butyrate	0.33	/	/
*α*-Cubebene	0.07	0.04	0.15
Copaene	0.20	0.18	/
*β*-Bourbon	0.31	0.71	0.75
Elemene	0.45	0.56	0.36
2-Ethyl-1,4-dimethyl-benzene	0.06	/	/
Caryophyllene	4.51	3.82	2.44
cis-*β*-Farnesene	0.11	/	/
a-Caryophyllene	0.28	0.26	0.15
Alloaromadendrene	/	/	0.38
*γ*-Muurolene	0.40	0.32	0.50
*β*-Cadinene	5.57	4.13	3.18
*γ*-Cadinene	/	/	0.18
*α*-Muurolene	/	/	0.52
*β*-Selinene	0.07	/	/
(+)-*δ*-Cadinene	0.84	0.51	0.75
*α*-elemol	3.54	3.03	1.87
Spathulenol	0.40	0.28	/
Caryophyllene oxide	0.99	1.77	1.03
*β*-Irisone	/	/	0.18
*γ*-Eudesmol	/	1.17	0.73
*β*-Eudesmol	2.10	1.49	/
*α*-Eudesmol	/	0.93	0.63
Benzyl benzoate	/	0.04	/
Copaene	/	/	0.11
PMA hydrochloride	0.07	/	/
Phytone	0.35	0.83	0.32
D(+)-Carvone	0.04	/	/
n-Hexadecanoic acid	1.24	1.92	/
phytol	0.24	0.09	0.11
(Z,Z)-9,12-Octadecadienoic acid	0.15	/	/
Heneicosane	/	0.03	/
Eicosane	/	0.06	/

/ represents compound not detected.
